# Muscle Responses to Passive Joint Movements in Infants During the First Year of Life

**DOI:** 10.3389/fphys.2019.01158

**Published:** 2019-09-13

**Authors:** Irina A. Solopova, Dmitry S. Zhvansky, Irina Y. Dolinskaya, Elena S. Keshishian, Victor A. Selionov, Francesca Sylos-Labini, Francesco Lacquaniti, Yury Ivanenko

**Affiliations:** ^1^Laboratory of Neurobiology of Motor Control, Institute for Information Transmission Problems, Moscow, Russia; ^2^Moscow Research Institute of Clinical Pediatrics of Russian Federation, Moscow, Russia; ^3^Laboratory of Neuromotor Physiology, Fondazione Santa Lucia, Rome, Italy; ^4^Department of Systems Medicine and Center of Space Biomedicine, University of Rome Tor Vergata, Rome, Italy

**Keywords:** early development, passive movements, shortening reaction, stretch response, muscle tone, infants

## Abstract

Muscle tone represents one of the important concepts for characterizing changes in the state of the developing nervous system. It can be manifested in the level of activity of flexors and extensors and in muscle reactions to its passive stretching (StR) or shortening (ShR). Here we investigated such reactions in a cohort of healthy infants aged from 2 weeks to 12 months. We examined the presence and the characteristics of StR and ShR during slow passive cyclic flexion/extension movements (T~3 s) in the hip, knee, ankle, and elbow joints while awake infants were in the supine position. The results showed that most infants demonstrated prominent ShRs in response to passive joint rotations, although the StR was observed more frequently, suggesting that the ShR is an important component of adaptive motor behavior already at an early developmental stage. Interestingly, the occurrence of both StR and ShR in most muscles significantly decreased throughout the first year of life. Passive cyclic flexion/extension movements could also evoke rhythmic muscle responses in other joints or in the contralateral limb, however, such responses were predominantly observed in younger infants (<6 months). A noticeable manifestation of muscle reactions at an early developmental stage, along with spontaneous motor activity in this period of life, may reflect the processes underlying a formation of appropriate muscle tone and the self-organization of neural circuits. A substantial reduction of ipsilateral and contralateral muscle responses to passive movements with age is consistent with the idea of a functional reorganization of the motor circuitry during early development.

## Introduction

The notion of muscle tone has long been used to account for the mechanical properties of skeletal muscles at rest, in the limbs and along the whole body axis, and their disturbances in various pathologies (Martin, 1967). It is also important for characterizing changes in the state of the developing nervous system along with maturation of the motor circuitry and adjustments in habitual postures. It has been previously suggested that the development of muscle tone of newborns is intimately related to CNS maturation, as well as the clinical evaluation of muscle tone can also be a reliable indicator of the neurological status in the neonatal period (Amiel-Tison et al., 1999; da Silva and Nunes, 2005).

An important issue is an evaluation and definition of muscle tone (Ivanenko and Gurfinkel, 2018), which is traditionally linked to the activity level of muscle. In clinical practice, changes in tonus are typically measured by the extent of the muscle resistance to stretch (StR). However, muscle length changes may also evoke involuntary shortening reactions (ShR), responsible for “compliant” posture behavior (Forster, 1927; Cacciatore et al., 2014), or elicit postural adjustments of distant muscles not being primarily stretched (Gurfinkel et al., 1989; Teulier et al., 2011). In this respect, Bernstein (1940) provided a more functional interpretation of muscle tone, as the degree of readiness for movement related to movement as a state is related to an action, or as a precondition is related to an effect. Indeed, impairments in muscle tone may reflect changes in the physiological state of the spinal and supraspinal circuitries (Courtine et al., 2009; Edgerton and Roy, 2012; Selionov et al., 2013) and affect movement performance (Martin, 1967; Ivanenko et al., 2013b).

Shortening reaction was likely first described by Westphal (1880) and later it was reported in the numerous studies on both healthy (Safronov, 1970; Walsh, 1976; Katz and Rondot, 1978; Gurfinkel et al., 2006) and neurologically impaired individuals (Landau et al., 1966; Esslen, 1969; Andrews et al., 1972; Lee and Tatton, 1975; Angel, 1982; Miscio et al., 2001; Xia et al., 2009; Selionov et al., 2013). The first views on the functional role of the ShR belong to Forster (1927), who considered it as an adaptation reflex of muscle to its length. However, while the stretch/tendon reflexes (responses to tendon taps or vibration, Teulier et al., 2011) and the flexion reflex responses to innocuous stimulation (Andrews and Fitzgerald, 1999) have been previously reported in human infants, to our knowledge, the occurrence and the characteristics of the ShR have never been systematically investigated. In particular, it is unclear whether the ShR is determined in early development or this type of adaptive muscle behavior evolves later. Furthermore, given irradiation of responses to mechanical or electrical stimulation to distant muscles and their dependence on age in human infants (O'Sullivan et al., 1991; Myklebust and Gottlieb, 1993; Leonard et al., 1995; Andrews and Fitzgerald, 1999; Teulier et al., 2011), it is of interest to study the characteristics of evoked muscle activity during externally generated joint movement in the distant muscles of ipsilateral and contralateral limb joints.

Therefore, the aim of this study was to characterize muscle responses in infants during imposed passive angular displacements in the lower and upper limb joints and their dependence on age. To this end, we examined the presence and the characteristics of StR and ShR during slow cyclic passive flexion/extension movements in the hip, knee, ankle and elbow joints with approximately the same duration across all infants (0.5–12 months) and at angular velocities belonging to a natural repertoire of spontaneous or stepping movements in infants (Hadders-Algra et al., 1992; Kanemaru et al., 2012; Ivanenko et al., 2013a; Yang et al., 2015; Forma et al., 2018; La Scaleia et al., 2018). The results are discussed in the context of a functional reorganization and maturation of the motor circuitry during early development.

## Materials and Methods

### Participants

Participants were 54 healthy full-term infants, 23 females and 31 males, from 0.5 to 12 months postnatal age. Data from five additional infants were discarded for analysis due to unquiet behavior of infants that could not be calmed before the next trials. Eight infants participated in the study several times (one infant four times, four infants three times, five infants two times), the interval between the experimental sessions was 2–6 months and these infants were included in different groups, respectively, so that we performed 70 registrations total (Table 1). Since one of the aims of this study was to examine the effect of age, the infants were assigned to different age groups (0.5–3, 3–6, 6–9, and 9–12 mo, Table 1). Inclusion criteria for infants were: Apgar score >7 at 1 and 5 min, uneventful delivery and perinatal history, no known neurological or musculoskeletal pathology physical, and gestational age >38 week [group mean ± SD: 39.5 ± 0.6 weeks]. The exclusion criteria for the study were: congenital malformations, genetic and metabolic diseases, ongoing mechanical ventilation therapy. All infants were recorded at the Moscow Research Institute of Clinical Pediatrics. A parent for the child provided informed written consent to participate in the study. The protocol had been approved by the Ethics Committee of the Moscow Research Institute of Clinical Pediatrics (protocol n.14/18), and was conducted in accordance with the Declaration of Helsinki for experiments on humans.

**Table 1:** The total number of flexion/extension movements recorded in different joints in infants of each age group (the data for the right and left limb joint movements were summed up).

	**Group 1 (0.5-3 mo)**		**Group 2 (3-6 mo)**		**Group 3 (6-9 mo)**		**Group 4 (9-12 mo)**	
	**N infants**	**N movements**	**N infants**	**N movements**	**N infants**	**N movements**	**N infants**	**N movements**
*Hip flexion*	19	203 (5.5 ± 1.7)	25	241 (5.2 ± 1.2)	13	116 (4.5 ± 1.1)	11	110 (5.1 ± 1.3)
*Hip extension*		201 (5.5 ± 1.6)		243 (5.3 ± 1.1)		114 (4.5 ± 1.3)		103 (4.9 ± 1.3)
*Knee flexion*	19	198 (5.0 ± 1.7)	27	239 (4.6 ± 1.3)	13	100 (4.1 ± 1.2)	11	97 (5.0 ± 1.2)
*Knee extension*		195 (5.3 ± 1.8)		240 (4.8 ± 1.2)		100 (4.1 ± 1.3)		92 (4.3 ± 1.2)
*Dorsiflexion*	15	121 (3.9 ± 1.8)	22	223 (5.3 ± 1.9)	11	95 (4.3 ± 1.9)	10	89 (4.2 ± 1.3)
*Plantarflexion*		126 (4.2 ± 1.6)		221 (5.2 ± 2.0)		95 (4.4 ± 2.0)		91 (4.3 ± 1.2)
*Elbow flexion*	19	164 (4.4 ± 1.3)	27	237 (4.6 ± 1.2)	13	113 (4.2 ± 1.2)	11	103(4.7 ± 1.2)
*Elbow extension*		157 (4.1 ± 1.2)		216 (4.4 ± 1.3)		108 (4.1 ± 1.0)		95(4.3 ± 1.2)

*Mean (±SD across infants) values are also indicated in parentheses*.

### Experimental Procedure

**Figure 1:**
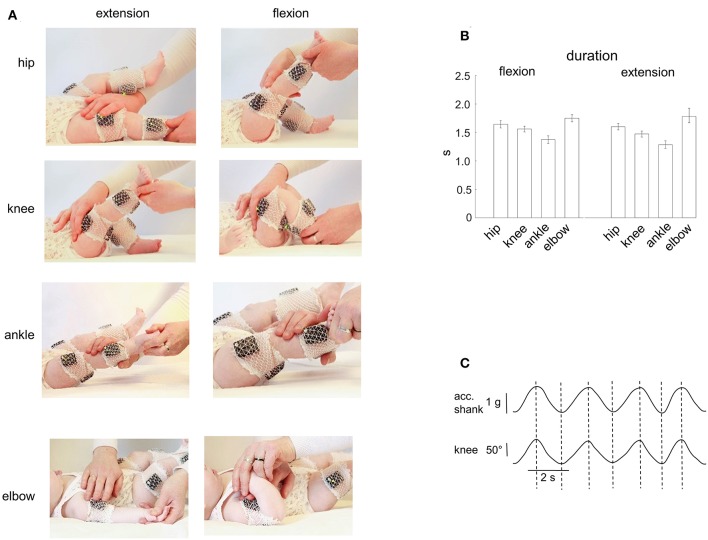
Passive flexion/extension movements in different joints in infants. **(A)** illustration of flexion-extension movements in the hip, knee, ankle, and elbow joints. EMG activity and IMU (acceleration) measurements of segment motion were registered using the Trigno Wireless EMG System (Delsys, Boston, MA). **(B)** mean duration (±95% confidence interval across all infants) of the flexion and extension movement duration in four joints (the data for the right and left limb movements were pooled together). **(C)** simultaneous registration of the acceleration (using the Trigno system IMU measurements, the acceleration signal is fused with the gravity vector) and the knee angle (using a goniometer) of passive flexion/extension cyclic movements. Note the correspondence (vertical dashed lines refer to the peaks of the knee joint angle) of the two signals.

The infants were tested in a quiet room. Passive flexion/extension movements were performed in the knee, hip, ankle and elbow joints (Figure 1A). All infants were tested when awake and alert. Infants were comfortable placed supine on a standard medical couch. If the child was quiet and friendly, then the parent(s) simply watched the recordings. If a child was too active or not very attuned to contact, then an additional motivation, such as toys, video for babies, or communication with parents, was used to achieve the most relaxed state of the baby. The same experimenter performed passive movements in all infants. We always started with movements in the knee or hip joint and then in the ankle and elbow joints. The limb segment orientations and the ranges of angular motion are illustrated for one child in Figure 1A. For hip joint movements, the initial position corresponded to an extended leg and the experimenter moved the whole limb around the hip joint (the hip angular excursion was ~60–70°). For knee joint movements, the experimenter maintained a more proximal (thigh) leg segment stationary by one hand (the hip angle was ~135°, the initial knee angle was also ~135°) while slowly changing shank orientation by another hand (the range of knee angular motion was ~120°). For ankle joint movements, the leg was about extended (the hip joint angle was ~165–175°, the knee joint angle was ~160°) and the range of ankle joint movements was ~30–40°. For elbow joint movements, the arm lay on the coach and the range of elbow joint flexion/extension movements was ~120–140° (Figure 1A). Movements where the baby spun, strained a limb (resisted to the experimenter), or tried to turn or raise the head, were excluded. Generally, 4–7 passive flexion/extension cyclic movements were performed for each joint, and we successfully recorded passive movements in almost all joints (Table 1). Passive movements were recorded in both right and left limb joints. The mean duration of movements in each joint is shown in Figure 1B. The total number of flexion/extension movements in each joint, selected for analysis, is shown in Table 1. The experimental session lasted ~20–30 min (including placement of EMG electrodes).

### Data Recording

EMG activities were recorded bilaterally using the Trigno Wireless EMG System (Delsys Inc., Boston, MA), bandwidth of 20–450 Hz, overall gain of 1,000, sampling rate 1,926 Hz. We recorded bilaterally from the following muscles: rectus femoris (RF), biceps femoris (BF), tibialis anterior (TA), gastrocnemius lateralis (GL), biceps brachii (BB), and triceps brachii (TB). The size of the Trigno bar EMG electrodes (5 mm) was relatively small in order to minimize a crosstalk. The Trigno EMG sensors also contained the IMU sensors so that we recorded the acceleration signals in the RF, TA, and BB sensors as well (at 148.1 Hz) to characterize movements in the hip and knee joints (see below). The skin was cleaned and rubbed slightly with alcohol before placing the electrodes. All movements of infants were recorded by a digital video camera (Panasonic HC-V760 EE-κ, 1920 × 1080 pixels, 50 frames/s). EMG and video recordings were synchronized.

### Definition of Flexion/Extension Movement Onsets and Durations

**Figure 2:**
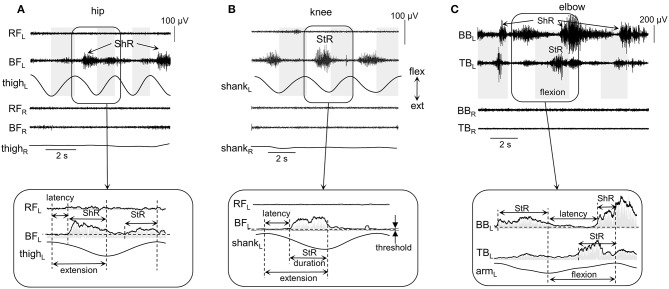
Examples of shortening (ShR) and stretch (StR) responses during passive movements in the knee **(A)**, hip **(B)**, and elbow **(C)** joints in three infants (4.5, 3, and 1.5 mo, respectively). The duration of the flexion phases is marked by the gray shaded areas (for hip and knee movements, it was defined from the thigh and shank acceleration signals, respectively; for the elbow joint, it was determined from video recordings, see Methods). RF, rectus femoris; BF, biceps femoris; BB, biceps brachii; TB, triceps brachii; R, right; L, left. Lower panels (inserts) refer to the EMG envelopes and the calculation of corresponding StR and ShR durations (when EMG activity exceeds the threshold, see Methods). Note various manifestations of muscle responses in these examples (and also minimal if any crosstalk in the recorded antagonist muscles): the presence of StR and ShR in only one muscle in **(A)** and **(B)**, and in both antagonist muscles in **(C)**.

The recordings of passive joint movements were first examined to determine the onset and termination of flexion and extension movements in all joints. For hip and knee joint movements, we used the Trigno system IMU acceleration signals (Figure 2A) to define the onset of motion of thigh and shank segments, respectively (and we checked it also by video recordings). The acceleration signals were detrended and low-pass filtered (0.05–1 Hz band-pass, fourth-order Butterworth filter). For elbow and ankle joint, passive movements were mainly analyzed using video recordings since the arm and shank segments (in which we recorded the EMG and IMU signals) were essentially motionless during movements of the distal segments (forearm and foot, respectively, Figure 1A).

Potential inaccuracy in the time interval of flexion and extension movements was about 50 ms and was checked for both measurements. For knee and hip joints, in a separate experiment, we recorded passive movements in these joints in one infant using simultaneously two different systems: (a) IMU signals of the Trigno system, and (b) goniometers (Figure 1C). The IMU acceleration signal is essentially fused with the gravity vector (especially during relatively slow movements like in our recordings) and thus largely reflects the tilt of thigh and shank segments relative to gravity. We found that the timings estimated independently by the two recording systems were very similar: the mean absolute difference between the onset of flexion/extension phases obtained by the two methods was on average 60 ± 30 ms (the data for the hip and knee joint movements were pooled together). For elbow and ankle joints, two experimenters (I.S. and D.Z.) independently examined the original video recordings, and we retained for further analyses the average timing. In general, in most cases both experimenters agreed and the correspondence was relatively good: the difference between the timing of the onset of flexion/extension phases obtained by the two experimenters was about 1–3 frames of video recordings (on average, 33 ± 20 ms). Note that the 30–60 ms error corresponds to <3–5% error (since the duration of movements was ~1.5 s, Figure 1B).

### EMG Data Analysis

Each flexion and extension phase was analyzed separately. To estimate the EMG envelopes, the interference EMG signals were rectified and smoothed by a sliding 20-ms window using the root mean square (RMS) averaging method (Merletti and Parker, 2004). To characterize muscle responses to passive movements in the hip, knee, ankle, and elbow joints, we calculated the onset (latency), duration, amplitude, and occurrence of StR and ShR.

The following criteria were used to define the onset (latency) and duration of muscle responses (Figure 2). First, the baseline level of muscle activity was assessed by calculating the mean EMG level of all signal fragments of at least 0.5 s in length, for which the average EMG amplitude was no more than 1.2 times larger than the minimum level throughout the trial (assuming that it represents or is close to the level of noise in the individual muscle recordings). Then, we determined the EMG responses according to the following algorithm: (1) we detected the periods of EMG activity exceeding the baseline level at least twice and not <30 ms in length; (2) if the interval between them was <50 ms, then they were merged into a single muscle response. If muscle activity continued after a change in the direction of movement (e.g., from flexion to extension), then the duration was determined only from the instant it started until the end of this phase. Such periods of EMG activity were considered as StR, if their onset was in the stretching phase of the corresponding muscle and the duration exceeded 100 ms. Similarly, muscle activity was considered as ShR, if its onset occurred during the shortening phase and the duration exceeded 100 ms. Figure 2 illustrates examples of StR and ShR observed in only one muscle (as in A and B) or both antagonist muscles (C) and corresponding calculated latencies and durations. The latency was defined as the delay of StR and ShR onsets relative to the onset of extension and flexion, respectively. StR and ShR latencies and durations were normalized and expressed in percent of extension and flexion durations, respectively.

The occurrence of StR and ShR for each muscle, joint and for each infant was evaluated as the percentage of movements, in which these responses were present, relative to the total number of extension or flexion movements, respectively (the data for both left and right limbs were pooled together). The magnitude of StR and ShR was assessed as the mean EMG activity (in μV) during the defined interval of the corresponding muscle response.

### Statistics

The experimental data mostly did not meet the normal distribution criteria (Shapiro-Wilk's W-test, *p* < 0.05), therefore, non-parametric statistics were used for data analysis. Descriptive statistics included means and 95% confidence intervals of mean (except for Table 1). For comparison of independent samples, we used the Kruskal-Wallis test and the Mann-Whitney *U*-test with Holm-Bonferroni correction for the *post hoc* analysis (we compared movements in different joints, during flexion/extension, and in different age groups). For comparison of two dependent samples, we used the Wilcoxon matched pair test. Proportions difference *Z*-test was used to compare two independent proportions. The degree of relationship between StR and ShR amplitudes and the age of infants was assessed using Spearman rank-order correlation. The level of statistical significance was set at 0.05.

## Results

### General Characteristics of Passive Movements Induced in Different Joints in Infants

Figure 1A illustrates an example of flexion-extension movements in the hip, knee, ankle, and elbow joints in one infant. Passive movements were recorded in both right and left limb joints, were periodic (generally, 4–7 consecutive flexion/extension cycles) and relatively slow. The contralateral limb was not moved by the experimenter and the infants maintained it motionless in most cases (see, for instance, Figure 2), although in some cases we observed muscle responses in the contralateral limb (see below the last section of the Results “*Muscle responses in other joints of the ipsilateral and contralateral limbs*”). Even though the passive rotations were manually performed, the same experimenter executed measurements in all subjects in an approximately similar manner. The mean duration of flexion and extension movements in each joint was about 1.5 s in all joints (Figure 1B), being slightly shorter for the ankle joint (likely because the smaller range of angular motion). Infants completed the experimental procedures almost in all joints and, on average, we recorded the same number (~5) of flexion and extension movements in each joint, limb (left and right), and group of infants (Table 1).

First, we show the experimental data and the characteristics of muscle reactions in the joints being primarily rotated, and in the last section we report the incidents of induced muscle responses in other joints of the ipsilateral and contralateral limbs.

### Types of Muscle Responses to Passive Movements

On the whole, during imposed passive angular movements, all infants showed EMG responses in the muscles of the corresponding joints. Figure 2 illustrates examples of neuromuscular responses to passive angular joint movements in the lower (A,B) and upper (C) limbs. Major types of reactions to passive flexion/extension movements in infants included: predominantly ShR (Figure 2A) or StR (Figure 2B) in one muscle with no contemporaneous responses in the antagonist muscle, or more complex responses including the presence of StR and ShR in both antagonist muscles (Figure 2C).

Thus, muscle responses were not restricted to the muscle lengthening reactions, however, we often observed prominent EMG activity during muscle shortening as well. Because most of our recorded muscles were bi-articular, the same muscle could be activated during movements in different joints. For instance, for the hip joint movements, ShR was observed in RF during flexion and in BF during extension, while, for the knee joint movements, ShR was detected in RF during extension and in BF during flexion. For the ankle joints, ShR was observed in in TA during dorsiflexion and in GL during plantarflexion. For the elbow joint, ShR was prominent in BB during flexion and in TB during elbow joint extension. Muscle responses could be rather variable from cycle to cycle (Figures 1A,C). For instance, if ShR appeared in the first cycle, it could be observed in all other cycles, but it could also be evoked not in all cycles (Figure 2A). The irregular incidence was characteristic for the ShR as well, consistent with a generally high “within infants” and “between infants” variability in muscle responses to mechanical stimulation such as tendon taps or vibration (Teulier et al., 2011). Simultaneous occurrence of both StR and ShR in antagonist muscles was generally characterized by the early appearance of StR in one muscle and then ShR in the antagonist (Figure 2C). The latter type of muscle responses (co-existence of both StR and ShR) was observed on average in 22% of the total number of movements in all joints. Below we report the detailed characteristics of StR and ShR (occurrence in different joints, latency, age dependence).

### Comparison of StR and ShR in the Muscles of the Joints Being Rotated

**Figure 3:**
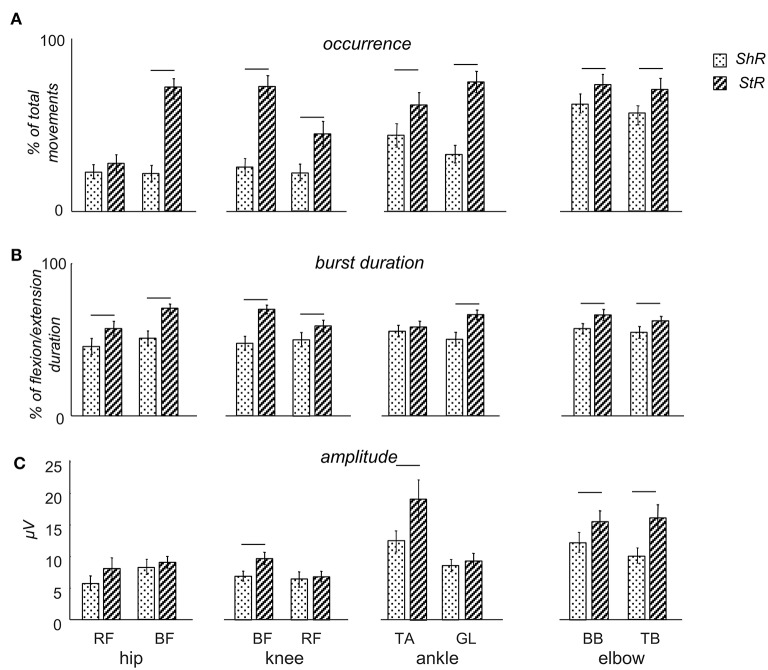
Characteristics of muscle responses (StR and ShR) to passive flexion/extension movements in different joints in infants. **(A)** occurrence of StR and ShR (the number of movements in which these responses were present, as the percentage of total number of extension or flexion movements, respectively). Data are presented as mean±95% confidence interval. **(B)** StR and ShR durations (expressed in percent of extension or flexion movement duration, respectively). **(C)** mean EMG activity (μV) of StR and ShR. RF – rectus femoris, BF – biceps femoris, TA – tibialis anterior, LG – gastrocnemius lateralis, BB – biceps brachii, TB – triceps brachii. The data for all infants were pooled together. Horizontal lines denote significant differences between StR and ShR. Note overall the higher occurrence and larger durations of StR than ShR.

Figure 3A illustrates the occurrence of StR and ShR in all joints and muscles during passive movements in the corresponding joints (the data for all infants were pooled together). For all muscles, the StR was observed more often than ShR (*p* < 0.01, Wilcoxon *T*-test with multiple testing Holm correction). However, ShR was significantly more pronounced in the upper limb than lower limb muscles (*p* < 0.01 Wilcoxon *T*-test). Interestingly, the occurrence of ShR in the same bi-articular leg muscles (RF and BF) was similar during movements in both hip and knee joints (Figure 3A). We did not find significant gender differences in the occurrence of ShR (*p* > 0.2 for all muscles and joints, Wilcoxon *T*-test). For StR, there were also no differences for most muscles (*p* > 0.01) except for BF (in the latter case, StR was observed more often in females, *p* = 0.01).

Table 2 shows the latencies of muscle responses. As a rule, ShR also began later than StR with respect to the onset of the flexion/extension phase in all joints (*p* < 0.04 for all muscles, Wilcoxon *T*-test, Table 2), although the latencies of StR and ShR were similar for all joints (on average, 460 and 630 ms, respectively, or 30 and 40% of the flexion/extension phase). For that reason, the muscle response was also shorter for ShR than for StR for most muscles except for TA (Figure 3B). In addition, the amplitude of StR (in μV) was larger for arm muscles (*p* < 0.0001, Wilcoxon *T*-test), knee flexors (*p* < 0.0001, Wilcoxon *T*-test) and ankle dorsiflexors (*p* < 0.02, Wilcoxon *T*-test) (Figure 3C).

**Table 2:** Mean latencies of StR and ShR in different muscles expressed in percent of corresponding flexion/extension phases (±95% confidence interval across all infants).

		**StR, % of flexion/extension**	**ShR, % of flexion/extension**	***p*-value**
Hip	RF	34 ± 4 (587 ± 69 ms)	41 ± 5 (708 ± 86 ms)	0.23
	BF	27 ± 3 (466 ± 52 ms)	42 ± 5 (684 ± 86 ms)	0.000001*
Knee	BF	26 ± 3 (416 ± 48 ms)	42 ± 5 (672 ± 80 ms)	0.000001*
	RF	28 ± 3 (431 ± 46 ms)	42 ± 5 (648 ± 77 ms)	0.0001*
Ankle	TA	35 ± 4 (478 ± 55 ms)	38 ± 3 (519 ± 41 ms)	0.24
	GL	31 ± 3 (400 ± 39 ms)	38 ± 5 (491 ± 65 ms)	0.04*
Elbow	BB	20 ± 3 (370 ± 56 ms)	38 ± 3 (697 ± 56 ms)	0.000001*
	TB	32 ± 3 (580 ± 54 ms)	36 ± 4 (653 ± 73 ms)	0.04*

*In parentheses, we also indicated the mean latencies in ms. Asterisks denote significant differences (p-values are also indicated, Wilcoxon T-test with multiple testing Holm correction) between StR and ShR latencies*.

It is also worth noting that, while both StR and ShR were often observed in infants during passive movements (Figure 3A) and while co-activation of antagonist muscles may often take place in infants during spontaneous or stepping movements (Thelen and Fisher, 1983; Teulier et al., 2012; Sylos-Labini et al., 2017), the presence or absence of ShR was not strictly related to the incidence of StR: these reactions could be well observed separately (Figures 2A,B). Furthermore, the correlation (r) between EMG envelopes of StR and the corresponding concurrent EMG activity of the antagonist muscle was on average rather low (~0.21): *r* = 0.05 for hip movements, 0.03 for knee movements, 0.35 for ankle movements, and 0.33 elbow movements. Only in a few cases we observed high correlations (>0.5), possibly potentially related to crosstalk since the ratio of EMG amplitudes of the antagonist muscles was >5, however, the percentage of such cases was <3% of the total number of ShR/StR (we discarded this data). Finally, the relationship between the occurrence of ShR and StR in the antagonist muscles for each direction (flexion or extension) of movement in all joints was assessed by the Spearman rank-order correlation coefficient, and none of the correlations was significant [the average value of the correlation coefficient was 0.07 ± 0.16 (mean ± SD)], meaning that, in some infants, predominantly ShR was observed during joint flexion (or extension), in some infants—predominantly StR, and in the others—both of them. Overall, both types of muscle responses could be frequently observed in infants (Figure 3), suggesting that ShR belongs to a natural repertoire of “compliant” motor behavior already at an early developmental stage.

### Effect of Age

**Figure 4:**
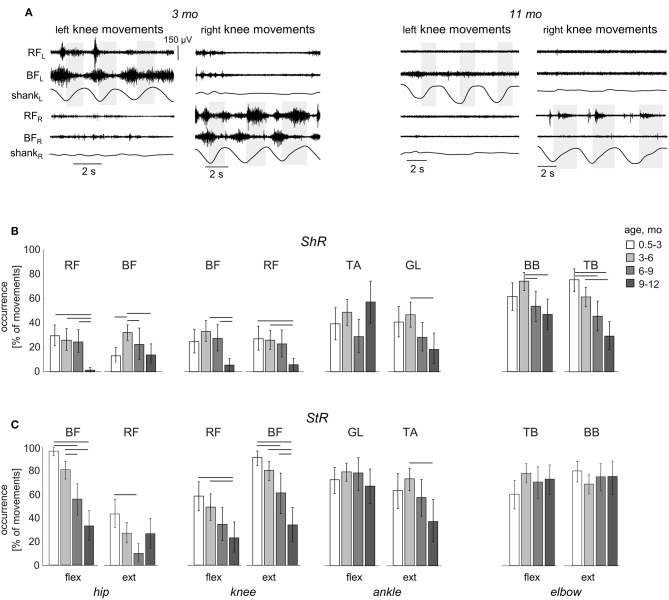
Effect of age on the occurrence of ShR and StR in infants. **(A)** example of muscle responses during knee flexion/extension movements in one infant recorded twice: at 3 mo (left panels) and 11 mo (right panels). The same format as in Figure 2. Note prominent StR and ShR in the thigh muscles at 3 mo and a lack of muscle responses at 11 mo. **(B,C)** occurrence of StR and ShR (as the percentage of total number of extension or flexion movements, mean±95% confidence interval) during passive movements in the hip, knee, ankle, and elbow joints in different age groups of infants (0.5–3 mo, 3–6 mo, 6–9 mo, and 9–12 mo). Horizontal lines denote significant differences. Note a significant decrease of muscle responses during passive movements in most muscles with age.

**Figure 5:**
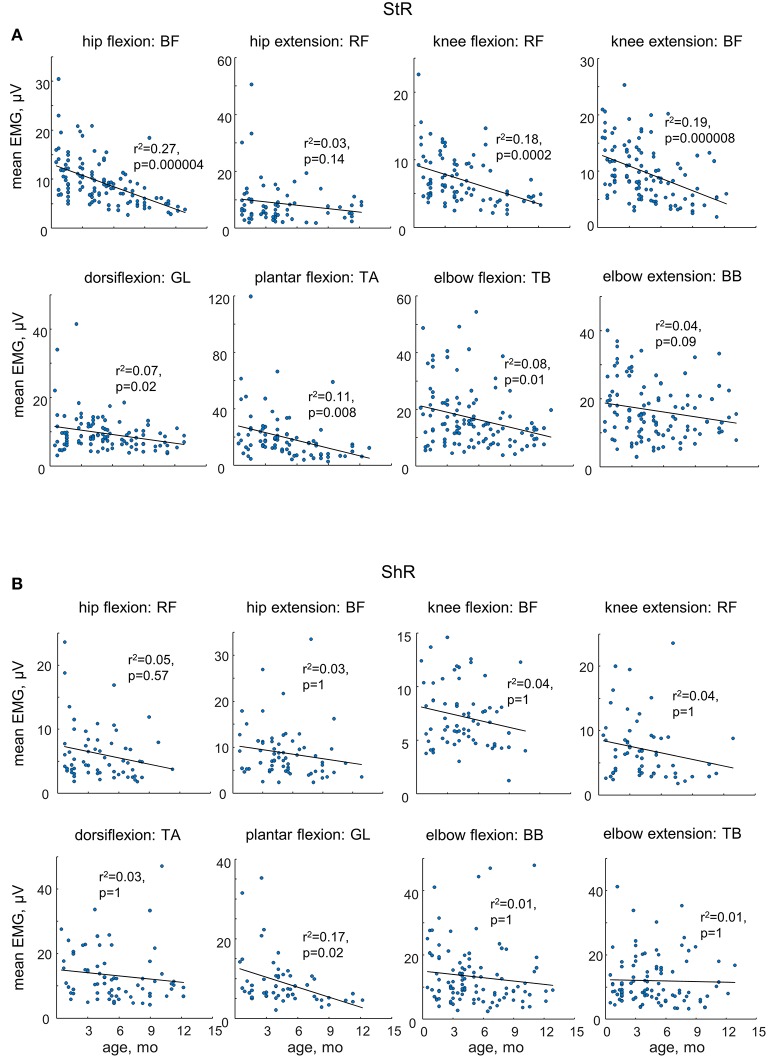
Mean EMG activity of ShR **(A)** and StR **(B)** in different joints in infants as a function of age. Each point represents the mean value for an individual infant. Linear regression lines with corresponding r and p values are reported. Note a decrease in the amplitude of StR in most muscles with age.

While muscle responses to passive movements were observed in all age groups, their occurrence significantly decreased with age. For instance, Figure 4A shows an example of muscle responses during knee flexion/extension movements in one infant recorded twice: at 3 mo (left panels) and 11 mo (right panels). Note prominent StR and ShR in the thigh muscles at 3 mo and a lack of most of them at 11 mo. We compared the occurrence of muscle responses in four age groups of infants during the first year of life (0.5–3, 3–6, 6–9, and 9–12 mo, Table 1). Figures 4B,C illustrate a significant decrease of muscle responses during passive movements in most joints/muscles with age (*p* < 0.03, *post hoc* Mann-Whitney *U*-test). We used non-normalized (in μV) EMG data to determine StR and ShR, However, even when we considerably increased the threshold for detecting the periods of muscle activity (three times exceeding the baseline level instead of two times, see Methods), the percentage of detected muscle responses decreased (by ~25%), however, the effect of age (Figures 4B,C) remained the same for most muscles. Furthermore, even though the amplitude of EMG activity in μV can be considered only as a qualitative parameter (due to potential differences in skin impedance across infants), the amplitude of EMG responses also tended to decrease with age in most muscles (see linear regression lines with corresponding *r*^2^ and *p*-values in Figure 5) during muscle lengthening though not during muscle shortening (except for GL). The age had no significant influence on StR and StR latencies (*p* > 0.1, effect of group, Kruskal-Wallis test).

### Muscle Responses in Other Joints of the Ipsilateral and Contralateral Limbs

**Figure 6:**
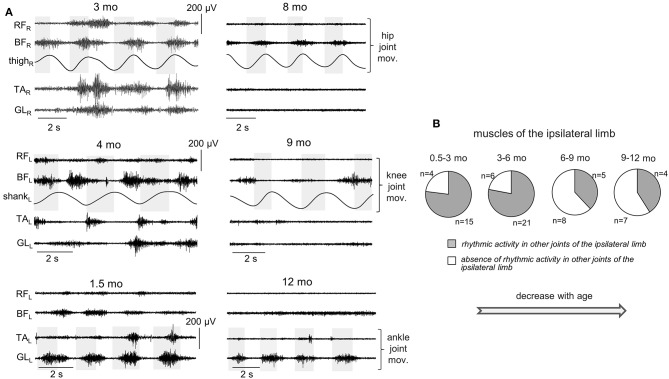
Occurrence of muscle responses in other joints (not being passively rotated) of the ipsilateral limb. **(A)** examples of rhythmic-like movements and EMG activity in the ipsilateral limb during passive flexion/extension movements in the hip, knee, and ankle joints in different infants (the age is indicated, same format as in Figure 2). **(B)** pie charts showing the percentage of infants (their number is also indicated) with the presence of rhythmic activity in the ipsilateral limb for four age groups. The data for all joints were pooled together (for each joint, see the data in Table 3). Note a decrement of muscle responses with age (in groups 3–4 with respect to groups 1–2).

**Figure 7:**
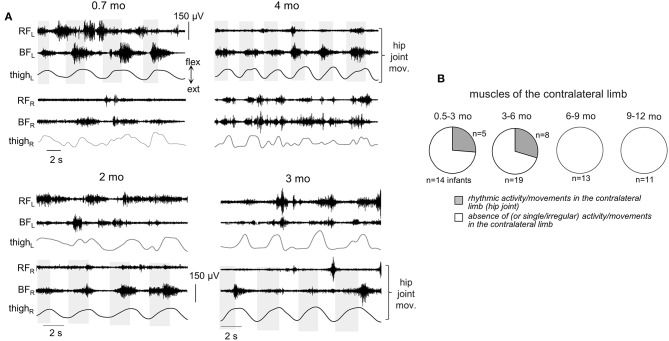
Occurrence of muscle responses in the contralateral limb. **(A)** examples of rhythmic-like movements and EMG activity in the contralateral limb during passive flexion/extension movements in the hip joint in different infants (the age is indicated, same format as in Figure 6). Upper panels—passive movements were induced in the left hip joint, lower panels—in the right hip joint. **(B)** pie charts showing the percentage of infants (their number is also indicated) with the presence of rhythmic activity in the contralateral limb for four age groups. Rhythmic muscle responses in the contralateral limb were mainly observed during passive movements in the hip joint. Note a lack of contralateral muscle responses in groups 3–4.

Since involvement or irradiation to distant muscles have been described from newborn infants to adults (Myklebust, 1990), we also looked for the incidence of consistent muscle responses in the joints not being primarily moved. In particular, Figures 6, 7 illustrate the occurrence of regular (rhythmic) muscle activity in the ipsilateral and contralateral limbs, respectively, in infants during passive movements in distant joints.

For ipsilateral influences, muscle responses in other joints were rather frequent (Figures 6A,B). During passive movements in the hip joints, EMG activity could be observed in both TA and GL muscles or only in one of these muscles. During movements in the ankle joint, muscle activity could be observed in BF and/or RF. During movements in the knee joint, we considered only the TA muscle, since GL is the bi-articular muscle and one cannot exclude its lengthening/shortening during knee joint rotations. In the upper limb, we could not perform such analysis since we did not record “distant” muscles in the forearm or wrist. Pie charts in Figure 6B show the percentage of infants with the presence of rhythmic activity in at least one or more joints of the ipsilateral limb. The incidence of such responses in distant muscles of the ipsilateral leg was the greatest in younger infants (group 1 and 2, age 0.5–6 mo). In older infants (6–12 mo), the examples of such activity were significantly (about twice) less frequent. The statistical analysis (*proportions difference Z-test* with multiple testing Holm correction) showed a greater percent of infants with ipsilateral influences in the group 1 than in the group 3 (*p* = 0.0007) or group 4 (*p* = 0.002). Analogously, for the group 2, it was greater that for the group 3 (*p* = 0.0003) or group 4 (*p* = 0.001).

Furthermore, Table 3 shows that a decrement of muscle responses with age was observed in the muscles of all joints. It is also worth noting that, in younger infants (0.5–6 mo), we often observed EMG activity in both antagonist muscles of the distal joint (TA and GL) during passive movements in the hip joint, and only in one distant muscle in a few cases. During movements in the ankle joint, the combined activity of hip muscles was observed in about 50% of cases (in remaining 50% of cases, only BF was activated). However, in older infants of group 3 (6–9 mo), the cooperative activation of TA and GL was observed less often (only in about 50% of cases). In the infants of group 4 (9–12 mo), typically only one of the distant muscles was activated.

**Table 3:** Occurrence of rhythmic activity in other joints (not being rotated) of the ipsilateral leg in each age group of infants (both the number of infants and percent of them in the parenthesis are indicated).

	**Group 1 (0.5-3 mo)**	**Group 2 (3-6 mo)**	**Group 3 (6-9 mo)**	**Group 4 (9-12 mo)**
Hip joint movements - activity in TA, GL	15/19 (79%)	20/25 (80%)	6/13 (46%)	4/11 (36%)
Knee joint movements - activity in TA	14/19 (73%)	19/27 (70%)	5/13 (38%)	5/11 (45%)
Ankle joint movements - activity in RF, BF	12/15 (80%)	18/22 (81%)	3/11 (27%)	4/10 (40%)

*Note a decrement of muscle responses with age (in groups 3–4 with respect to groups 1–2) in all joints*.

Finally, in younger infants (0.5–6 mo), there was a relatively frequent incidence of regular muscle responses in the contralateral limb (typically during passive rhythmic movements in the hip joint, Figure 7, see also Supplementary Movie 1), while in older infants such consistent contralateral influences were never recorded during passive movements in any joint.

## Discussion

To our knowledge, tonic muscle reactions in human infants have not been previously systematically investigated. The results showed frequent muscle responses (either separately or in both flexion and extension phases, Figure 2) during externally generated cyclic joint movements of the upper and lower limbs in infants of typical development between 0.5 and 12 months of age. Even though the ShR was observed less frequently than the StR (Figure 3), our data demonstrated that ShR belongs to a natural repertoire of “compliant” motor behavior already at a very early developmental stage (0.5–6 mo, Figure 4). Furthermore, cyclic passive movements in one joint could evoke repetitive motor responses in “remote” muscles (Figures 6, 7). Such responses were still recorded from 6 to 12 month-old infants but with less frequency than in infants <6 months of age (Figures 4, 6, 7), supporting the idea of maturation or reorganization of the sensorimotor circuitry in the process of early motor development.

### Limitations of the Study

When interpreting the current results, one should take into account the following limitations. First, an electromyographic technique (surface EMG recordings) that we used to evaluate muscle responses has some limitations. For instance, we used non-normalized EMG data and the infants have higher subcutaneous fat tissue, which might influence the EMG signal. Nevertheless, we determined StR and ShR relative to the individual baseline level of activity of each muscle (see Methods). Furthermore, even when we considerably increased the threshold (three times exceeding the baseline level) for detecting StR and ShR, the effect of age (Figures 4B,C) basically remained the same. Some amount of cross-talk can also be present in the surface EMG recordings in infants though the correlation with the corresponding concurrent EMG activity of the antagonist muscle was on average rather low, as well as we often observed separate StR and ShR in the antagonist muscles (see Results). Moreover, while some influences of the cross-talk on the muscle response characteristics could not be excluded, they cannot account for muscle responses in distant muscles or in the contralateral limb (Figures 6, 7).

Second, the 30–60 ms uncertainty in measured latencies (see Methods) also represents a limiting factor of our study. However, it is worth noting that we used relatively slow sinusoidal joint rotations and we reported long-latency (about 0.5 s, Table 2) muscle responses. Such long-latency responses are typical for tonic rather than phasic muscle contractions and can be observed in other conditions as well, for instance, during the tonic vibration reflex (delays of several seconds, Burke et al., 1972) and changes in the muscle length (Burke et al., 1978; Safronov, 1984; Xia et al., 2009; Selionov et al., 2013).

Finally, passive joint movements were provided manually and their variation could contribute to the variation in muscle responses. Nevertheless, even though the passive rotations were manually performed, the same experimenter executed measurements in all infants in an approximately similar manner and duration (~1.5 s in all joints and age groups, Figure 1B). Furthermore, manual movements are frequently used to assess muscle tone or spasticity in a clinical setting (Marinelli et al., 2013).

Variability in response magnitude (Figure 2), occurrence (Figure 3) and latencies (Table 2) might be related to the infant's behavioral state as well. It is worth stressing that results from our study are in line with the large variability in the stretch reflex magnitude previously observed during standardized muscle stimulation as well (O'Sullivan et al., 1991; Myklebust and Gottlieb, 1993; Leonard et al., 1995; Teulier et al., 2011). These studies examined tendon taps or stretch reflexes at short latencies and showed that diverse patterns of response could occur within an infant across repeated application of tendon taps (the force of the tendon tap was controlled for each individual infant), and across infants: a response in the stimulated muscle alone, a response in the antagonist muscle alone, a simultaneous response in both muscles or no response at all (Teulier et al., 2011). In part it could be mediated by oligosynaptic modulation of stretch reflexes (Lacquaniti et al., 1991) and by supraspinal influences (Feldman and Latash, 1982). Furthermore, the authors argued that such variability might be functional during infancy due to the lack of exposure to specific stimuli and tasks and, therefore, the need to learn to set stable feedback gain based on its use during functional movements and adaptive motor behaviors (Teulier et al., 2011). Whatever the exact neural pathways involved in modulation of muscle lengthening responses in infants, our data support their frequent incidence and variability, especially in younger infants (Figures 4, 5).

### Occurrence of StR and ShR in Human Infants

The expression of long-latency muscle responses to relatively slow stretch and shortening depends on the excitability level of the spinal and supraspinal networks, and can be considered as manifestation of muscle tone (Andrews et al., 1972; Lee and Tatton, 1975; Angel, 1982; Miscio et al., 2001; Xia et al., 2009; Gurfinkel et al., 2011; Selionov et al., 2013).

Interestingly, motor responses in infants were not restricted to muscle lengthening, and both types of muscle reactions (StR and ShR) could be observed either separately or within the same flexion or extension movement (Figure 2). The StR was observed more often than the ShR (Figure 3A), with shorter latencies (Table 2) and, accordingly, the longer duration (Figure 3B). Nevertheless, the occurrence of ShR was also frequent, especially in the upper limb muscles (Figure 3A), suggesting its important role during early infancy in learning and adaptive motor behavior. The role of group II muscle afferents (Mortimer and Webster, 1978), Golgi tendon organs (Safronov, 1984) and joint receptors (Katz and Rondot, 1978) in its generation have been previously discussed. The ShR has low thresholds (if one considers relatively low velocities of imposed joint rotations, Safronov, 1984) and is accompanied by a discharge of primary endings of muscle spindles, i.e., α-γ coactivation (Burke et al., 1978). While neural substrates of the ShR are not fully understood, its functional significance as an adaptation reflex of muscle to its length (Forster, 1927) is thought to be related to its role in the redistribution the muscle tone across antagonists (Miscio et al., 2001), providing spindle endings with a background discharge so that they can detect irregularities in the movement and initiate the appropriate reflex correction (Burke et al., 1978), and in compliant posture behavior (Safronov, 1970; Gurfinkel et al., 2006; Cacciatore et al., 2014). The latter aspect is especially important given its functional significance during everyday life activities and adaptive motor behavior.

Both types of muscle reactions (StR and ShR) could be observed either separately or within the same flexion or extension movement (Figure 2). Coexistence of StR and ShR should not be considered merely as co-activation, since they were often observed separately (Figure 2), as well as it is not a prerogative of motor responses only in infants. Indeed, during externally generated joint movement, the amount of the imposed resistance is a resultant of the interaction between the StR, ShR, and the spring-like force of stretched and shortening muscles. For instance, in healthy humans, the axial tone is modulated sensitively and dynamically, originating from both tonic lengthening and shortening reactions, and a similar type of control appears to exist for postural tone in the proximal muscles (Gurfinkel et al., 2006). Furthermore, paradoxical coexistence of prominent ShRs and rigidity have been previously documented in Parkinson Disease (Westphal, 1880; Andrews et al., 1972; Lee and Tatton, 1975; Berardelli et al., 1983; Xia et al., 2009). All these studies point to an important role of both stretch responses and shortening reactions in adaptive motor behavior, and both types of muscle responses are determined in early development (Figure 3).

### Effect of Age

In addition to the frequent incidence of motor responses to passive joint movements, our study revealed important age-related characteristics of these responses. First, both ShR and StR were still recorded from 6 to 12 month-old infants but with less frequency than in infants <6 months of age in most muscles (except for the ankle joint muscles, Figure 4). Their amplitude also tended to decrease, particularly for the StR (Figure 5). Second, cyclic passive movements in one joint could evoke repetitive motor responses in “remote” muscles of both ipsilateral (Figure 6) and contralateral (Figure 7) limbs, possibly also related to the so-called “irradiation” of responses to mechanical stimulation to distant muscles in infants (Myklebust and Gottlieb, 1993; Teulier et al., 2011). The contralateral effects can also be observed in adults in some conditions or pathologies (for instance, the phenomenon of “cross education” (Lee and Carroll, 2007) and “mirror movements” Carson, 2005; Kuo et al., 2018), although such effects generally take place during voluntary rather than passive movements.

Two distinct but not necessarily mutually exclusive hypotheses can be outlined that represent viable explanations for the effect of age. The first hypothesis suggests changes in excitability of spinal or supraspinal circuits, while the second hypothesis is related to functionally significant or task-relevant motor behavior. Indeed, the first months of life represent an extremely important phase of maturation of the central nervous system, processing of sensory information (von Hofsten, 2004; Anderson et al., 2019; Niutanen et al., 2019) and posture control (Jantz et al., 1997; Claxton et al., 2014; Duncan et al., 2018), which includes resistive, compliant and movement-related behavior. The latter aspect was well-formulated by Sherrington (1906): “posture follows movement like a shadow.” It is possible that the reason why the circuits are more excitable early on (Figures 4, 6, 7) is related to learning functionally appropriate dynamic and static postural tone, which can be assessed during changes in the muscle length and limb configuration. It may also be related to the self-organization of motor circuitries at an early developmental stage (Blankenship and Feller, 2010).

Thus, it is important to note that muscle responses were predominantly observed in younger infants (<6 months, Figures 4, 6, 7). The reported age-dependent manifestations of muscle tone, assessed by measuring a response to externally generated slow joint movements (Figures 4, 6, 7), substantiate other age-dependent changes in the functioning of the sensorimotor circuitry in early infancy. For instance, in neonates, the flexion reflex responses to innocuous stimulation are already present and the incidence of response to repeated mechanical stimulation decreases with increasing age (Andrews and Fitzgerald, 1999). Also, there is a reorganization of muscle activity during spontaneous movements in early infancy, such as an attenuation of bursts amplitude and a decrease of tonic background activity, suggesting that these changes may be attributable to a reduction of the sensitivity of the motor units due to spinal and supraspinal reorganization (Hadders-Algra et al., 1992). In addition, animal studies showed that there are developmental changes in the frequency firing and synchronization of motor neuron activity (Westerga and Gramsbergen, 1994; Tresch and Kiehn, 2002). Finally, there is a significant functional reorganization of the spinal locomotor output in toddlers at the onset of independent walking with respect to neonatal stepping, consisted in augmenting the number of basic muscle activation patterns and changes in the spinal maps of motor pool activity (Dominici et al., 2011; Ivanenko et al., 2013a; Lacquaniti et al., 2013).

### Clinical Implications

In addition to investigating the basic mechanisms of sensorimotor function maturation, there is growing interest in quantifying muscle activity and kinematic patterns in infants as predictive indicators of impaired motor development (Domellöf et al., 2007; Barbu-Roth et al., 2009; Hadders-Algra, 2014; Kanemaru et al., 2014; Ritterband-Rosenbaum et al., 2017). Future studies may thus focus on investigating a functional significance of early manifestations of premature muscle tone and its impairments in early sensorimotor disorders. Future experiments are also needed to clarify the contribution of spinal and supraspinal factors. The reported types of muscle responses to externally generated movements might be essential for the clinical evaluation of muscle tone as indicators of the neurological status (Esslen, 1969; Amiel-Tison et al., 1999; da Silva and Nunes, 2005), in conjunction with evaluation of the specific features of spontaneous movements, for instance, for predicting cerebral palsy in very young infants (Hadders-Algra, 2014; Kanemaru et al., 2014).

### General Conclusions

Taken together, the findings support a high degree of functionality of the sensorimotor circuitries but also a reorganization of the motor output during the first year of life. A dynamic “postural frame,” that is inherently incorporated in posture and movement coordination, may account for the resistive or compliant behavior of the body (Cacciatore et al., 2014), and the frequent occurrence of prominent ShR in infants as young as 0.5–3 mo suggests that it already belongs to an innate repertoire of compliant motor behavior. A noticeable manifestation of muscle reactions at an early developmental stage, along with spontaneous motor activity (Blankenship and Feller, 2010), may facilitate or reflect the processes underlying the self-organization of neural circuits at both spinal and supra-spinal levels in this period of life.

## Data Availability

The datasets generated for this study are available on request to the corresponding author.

## Ethics Statement

The studies involving human participants were reviewed and approved by Ethics Committee of the Moscow Research Institute of Clinical Pediatrics. Written informed consent to participate in this study was provided by the participants' legal guardian/next of kin.

## Author Contributions

IS and YI conceived and designed the experiments. IS, DZ, ID, EK, and VS performed the experiments. EK selected and examined the neurological state of infants. IS, DZ, ID, VS, and FS-L analyzed the data. IS, FL, and YI wrote the paper. All the authors made contributions in drafting the manuscript and interpreting the results and have approved the final version.

### Conflict of Interest Statement

The authors declare that the research was conducted in the absence of any commercial or financial relationships that could be construed as a potential conflict of interest.

## Supplementary Material

Supplementary Movie S1 Example of rhythmic motor responses in the contralateral limb during passive flexion/extension movements in the hip joint (2.8 mo infant).

## References

[B1] Amiel-Tison C. Maillard F. Lebrun F. Bréart G. Papiernik E. (1999). Neurological and physical maturation in normal growth singletons from 37 to 41 weeks' gestation. Early Hum. Dev. 54, 145–156. 10.1016/S0378-3782(98)00087-5 10213293

[B2] Anderson D. I. He M. Gutierrez P. Uchiyama I. Campos J. J. (2019). Do balance demands induce shifts in visual proprioception in crawling infants? Front. Psychol. 10:1388. 10.3389/fpsyg.2019.01388 31281282 PMC6595268

[B3] Andrews C. J. Burke D. Lance J. W. (1972). The response to muscle stretch and shortening in parkinsonian rigidity. Brain 95, 795–812. 10.1093/brain/95.4.795 4265061

[B4] Andrews K. Fitzgerald M. (1999). Cutaneous flexion reflex in human neonates: a quantitative study of threshold and stimulus-response characteristics after single and repeated stimuli. Dev. Med. Child Neurol. 41, 696–703. 10.1017/S0012162299001425 10587047

[B5] Angel R. W. (1982). Shortening reaction in patients with cerebellar ataxia. Ann. Neurol. 11, 272–278. 10.1002/ana.410110307 7092180

[B6] Barbu-Roth M. Anderson D. I. Desprès A. Provasi J. Cabrol D. Campos J. J. (2009). Neonatal stepping in relation to terrestrial optic flow. Child Dev. 80, 8–14. 10.1111/j.1467-8624.2008.01241.x 19236388 PMC2709813

[B7] Berardelli A. Sabra A.F. Hallett M. (1983). Physiological mechanisms of rigidity in Parkinson's disease. J Neurol Neurosurg Psychiatry. 46, 45–53. 10.1136/jnnp.46.1.45 6842199 PMC1027262

[B8] Bernstein N. A. (1940). “Studies of the biodynamics of walking, running and jumping. Moscow, Researches of the Central Scientific Institute of Physical Culture,” in Human Motor Actions. Bernstein Reassessed, eds Whiting H. T. A. (1984). (Amsterdam: North-Holland), 171–222. 10.1016/S0166-4115(08)61373-4

[B9] Blankenship A. G. Feller M. B. (2010). Mechanisms underlying spontaneous patterned activity in developing neural circuits. Nat. Rev. Neurosci. 11, 18–29. 10.1038/nrn2759 19953103 PMC2902252

[B10] Burke D. Andrews C. J. Lance J. W. (1972). Tonic vibration reflex in spasticity, Parkinson's disease, and normal subjects. J. Neurol. Neurosurg. Psychiatry 35, 477–486. 10.1136/jnnp.35.4.477 4261955 PMC494108

[B11] Burke D. Hagbarth K. E. Löfstedt L. (1978). Muscle spindle activity in man during shortening and lengthening contractions. J. Physiol. 277, 131–142. 10.1113/jphysiol.1978.sp012265 148511 PMC1282382

[B12] Cacciatore T. W. Mian O. S. Peters A. Day B. L. (2014). Neuromechanical interference of posture on movement: evidence from Alexander technique teachers rising from a chair. J. Neurophysiol. 112, 719–729. 10.1152/jn.00617.2013 25085609 PMC4122698

[B13] Carson R. G. (2005). Neural pathways mediating bilateral interactions between the upper limbs. Brain Res. Brain Res. Rev. 49, 641–662. 10.1016/j.brainresrev.2005.03.005 15904971

[B14] Claxton L. J. Strasser J. M. Leung E. J. Ryu J. H. O'Brien K. M. (2014). Sitting infants alter the magnitude and structure of postural sway when performing a manual goal-directed task. Dev. Psychobiol. 56, 1416–1422. 10.1002/dev.21211 24604626

[B15] Courtine G. Gerasimenko Y. van den Brand R. Yew A. Musienko P. Zhong H.. (2009). Transformation of nonfunctional spinal circuits into functional states after the loss of brain input. Nat. Neurosci. 12, 1333–1342. 10.1038/nn.2401 19767747 PMC2828944

[B16] da Silva E. S. Nunes M. L. (2005). The influence of gestational age and birth weight in the clinical assessment of the muscle tone of healthy term and preterm newborns. Arq. Neuropsiquiatr. 63, 956–962. 10.1590/S0004-282X2005000600010 16400412

[B17] Domellöf E. Rönnqvist L. Hopkins B. (2007). Functional asymmetries in the stepping response of the human newborn: a kinematic approach. Exp. Brain Res. 177, 324–335. 10.1007/s00221-006-0675-4 16951957

[B18] Dominici N. Ivanenko Y. P. Cappellini G. d'Avella A. Mondì V. Cicchese M.. (2011). Locomotor primitives in newborn babies and their development. Science 334, 997–999. 10.1126/science.1210617 22096202

[B19] Duncan K. Goodworth A. Da Costa C. S. N. Wininger M. Saavedra S. (2018). Parent handling of typical infants varies segmentally across development of postural control. Exp. Brain Res. 236, 645–654. 10.1007/s00221-017-5156-4 29285555 PMC6190590

[B20] Edgerton V. R. Roy R. R. (2012). A new age for rehabilitation. Eur. J. Phys. Rehabil. Med. 48, 99–109. 22407010

[B21] Esslen E. (1969). Stretch reflex and shortening reaction. A quantitative analysis of phasic and tonic stretch reflex in clinical states of spasticity and rigidity. Electroencephalogr. Clin. Neurophysiol. 27:719. 10.1016/0013-4694(69)91394-7 4187444

[B22] Feldman A. G. Latash M. L. (1982). Inversions of vibration-induced senso-motor events caused by supraspinal influences in man. Neurosci. Lett. 31, 147–151. 10.1016/0304-3940(82)90107-0 6982436

[B23] Forma V. Anderson D. I. Goffinet F. Barbu-Roth M. (2018). Effect of optic flows on newborn crawling. Dev. Psychobiol. 60, 497–510. 10.1002/dev.21634 29851061

[B24] Forster O. (1927). “Schlaffe und spastische lähmung,” in Handbuch der Normale und Pathologischen Physiologie, eds Bethe A. Bergman G. von Embden G. Ellinger A A. (Berlin: Springer, 893–972.

[B25] Gurfinkel V. Cacciatore T. W. Cordo P. Horak F. Nutt J. Skoss R. (2006). Postural muscle tone in the body axis of healthy humans. J. Neurophysiol. 96, 2678–2687. 10.1152/jn.00406.2006 16837660

[B26] Gurfinkel V. S. Cacciatore T. W. Cordo P. J. Horak F. B. (2011). Method to measure tone of axial and proximal muscle. J. Vis. Exp. 58, 3677 10.3791/3677 PMC3369643 22214974

[B27] Gurfinkel V. S. Levik I. u. S. Lebedev M. A. (1989). Immediate and remote postactivation effects in the human motor system. Neurophysiology 21, 343–351. 10.1007/BF01058224 2770916

[B28] Hadders-Algra M. (2014). Early diagnosis and early intervention in cerebral palsy. Front Neurol. 5:185. 10.3389/fneur.2014.00185 25309506 PMC4173665

[B29] Hadders-Algra M. Van Eykern L. A. Klip-Van den Nieuwendijk A. W. Prechtl H. F. (1992). Developmental course of general movements in early infancy. II. EMG correlates. Early Hum. Dev. 28, 231–251. 10.1016/0378-3782(92)90170-L 1592008

[B30] Ivanenko Y. Gurfinkel V. S. (2018). Human postural control. Front. Neurosci. 12:171. 10.3389/fnins.2018.00171 29615859 PMC5869197

[B31] Ivanenko Y. P. Dominici N. Cappellini G. Di Paolo A. Giannini C. Poppele R. E.. (2013a). Changes in the spinal segmental motor output for stepping during development from infant to adult. J. Neurosci. 33, 3025–3036a. 10.1523/JNEUROSCI.2722-12.2013 23407959 PMC6619203

[B32] Ivanenko Y. P. Wright W. G. St George R. J. Gurfinkel V. S. (2013b). Trunk orientation, stability, and quadrupedalism. Front. Neurol. 4:20. 10.3389/fneur.2013.00020 23504009 PMC3596858

[B33] Jantz J. W. Blosser C. D. Fruechting L. A. (1997). A motor milestone change noted with a change in sleep position. Arch. Pediatr. Adolesc. Med. 151, 565–568. 10.1001/archpedi.1997.02170430031006 9193239

[B34] Kanemaru N. Watanabe H. Kihara H. Nakano H. Nakamura T. Nakano J.. (2014). Jerky spontaneous movements at term age in preterm infants who later developed cerebral palsy. Early Hum. Dev. 90, 387–392. 10.1016/j.earlhumdev.2014.05.004 24951073

[B35] Kanemaru N. Watanabe H. Taga G. (2012). Increasing selectivity of interlimb coordination during spontaneous movements in 2- to 4-month-old infants. Exp. Brain Res. 218, 49–61. 10.1007/s00221-012-3001-3 22249434

[B36] Katz R. Rondot P. (1978). Muscle reaction to passive shortening in normal man. Electroencephalogr. Clin. Neurophysiol. 45, 90–99. 10.1016/0013-4694(78)90345-0 78826

[B37] Kuo H. C. Friel K. M. Gordon A. M. (2018). Neurophysiological mechanisms and functional impact of mirror movements in children with unilateral spastic cerebral palsy. Dev. Med. Child Neurol. 60, 155–161. 10.1111/dmcn.13524 28884806 PMC8331099

[B38] La Scaleia V. Ivanenko Y. Fabiano A. Sylos-Labini F. Cappellini G. Picone S.. (2018). Early manifestation of arm-leg coordination during stepping on a surface in human neonates. Exp. Brain Res. 236, 1105–1115. 10.1007/s00221-018-5201-y 29441470

[B39] Lacquaniti F. Borghese N. A. Carrozzo M. (1991). Transient reversal of the stretch reflex in human arm muscles. J. Neurophysiol. 66, 939–954. 10.1152/jn.1991.66.3.939 1753296

[B40] Lacquaniti F. Ivanenko Y. P. d'Avella A. Zelik K. E. Zago M. (2013). Evolutionary and developmental modules. Front. Comput. Neurosci. 7:61. 10.3389/fncom.2013.00061 23730285 PMC3656358

[B41] Landau W. M. Struppler A. Mehls O. (1966). A comparative electromyographic study of the reactions to passive movement in parkinsonism and in normal subjects. Neurology 16, 34–48. 10.1212/WNL.16.1.34 5948005

[B42] Lee M. Carroll T. J. (2007). Cross education: possible mechanisms for the contralateral effects of unilateral resistance training. Sports Med. 37, 1–14. 10.2165/00007256-200737010-00001 17190532

[B43] Lee R. G. Tatton W. G. (1975). Motor responses to sudden limb displacements in primates with specific CNS lesions and in human patients with motor system disorders. Can. J. Neurol. Sci. 2, 285–293. 10.1017/S0317167100020382 809129

[B44] Leonard C. T. Matsumoto T. Diedrich P. (1995). Human myotatic reflex development of the lower extremities. Early Hum. Dev. 43, 75–93. 10.1016/0378-3782(95)01669-T 8575354

[B45] Marinelli L. Trompetto C. Mori L. Vigo G. Traverso E. Colombano F.. (2013). Manual linear movements to assess spasticity in a clinical setting. PLoS ONE 8:e53627. 10.1371/journal.pone.0053627 23335966 PMC3546077

[B46] Martin J. P. (1967). The Basal Ganglia and Posture. London: Pitman Medical Publishing Co. Ltd.

[B47] Merletti R. Parker P. J. (2004). “Electromyography: physiology, engineering, and non-invasive applications,” in IEEE Press Series on Biomedical Engineering (New York, NY: Wiley).

[B48] Miscio G. Pisano F. Del Conte C. Pianca D. Colombo R. Schieppati M. (2001). The shortening reaction of forearm muscles: the influence of central set. Clin. Neurophysiol. 112, 884–894. 10.1016/S1388-2457(01)00468-0 11336906

[B49] Mortimer J. A. Webster D. D. (1978). “Relationships between quantitative measures of rigidity and tremor and the electromyographic responses to load perturbations in unselected normal subjects and Parkinson patients,” in Cerebral Motor Control in Man Long Loop Mechanisms. Progress in Clinical Neurophysiology, ed Desmedt J. E. (Basel: Karger), 342–360.

[B50] Myklebust B. M. (1990). A review of myotatic reflexes and the development of motor control and gait in infants and children: a special communication. Phys. Ther. 70, 188–203. 10.1093/ptj/70.3.188 2304976

[B51] Myklebust B. M. Gottlieb G. L. (1993). Development of the stretch reflex in the newborn: reciprocal excitation and reflex irradiation. Child Dev. 64, 1036–1045. 10.2307/1131325 8404255

[B52] Niutanen U. Harra T. Lano A. Metsäranta M. (2019). Systematic review of sensory processing in preterm children reveals abnormal sensory modulation, somatosensory processing and sensory-based motor processing. Acta Paediatr. 10.1111/apa.14953. [Epub ahead of print]. 31350861

[B53] O'Sullivan M. C. Eyre J. A. Miller S. (1991). Radiation of phasic stretch reflex in biceps brachii to muscles of the arm in man and its restriction during development. J. Physiol. 439, 529–543. 10.1113/jphysiol.1991.sp018680 1654417 PMC1180122

[B54] Ritterband-Rosenbaum A. Herskind A. Li X. Willerslev-Olsen M. Olsen M. D. Farmer S. F.. (2017). A critical period of corticomuscular and EMG-EMG coherence detection in healthy infants aged 9-25 weeks. J. Physiol. 595, 2699–2713. 10.1113/JP273090 28004392 PMC5390881

[B55] Safronov V. A. (1970). Regulation of muscle tone. Biofizika 15, 1103–1111. 5482664

[B56] Safronov V. A. (1984). Some properties of the response of muscle to passive shortening. Hum. Physiol. 10, 294–300. 6544723

[B57] Selionov V. A. Solopova I. A. Zhvansky D. S. Karabanov A. V. Chernikova L. A. Gurfinkel V. S.. (2013). Lack of non-voluntary stepping responses in Parkinson's disease. Neuroscience 235, 96–108. 10.1016/j.neuroscience.2012.12.064 23321538

[B58] Sherrington C. (1906). The integrative action of the nervous system. New York, NY: Charles Scribner's Sons.

[B59] Sylos-Labini F. Magnani S. Cappellini G. La Scaleia V. Fabiano A. Picone S. (2017). Foot placement characteristics and plantar pressure distribution patterns during stepping on ground in neonates. Front. Physiol. 8:784 10.3389/fphys.2017.00784 29066982 PMC5641324

[B60] Teulier C. Sansom J. K. Muraszko K. Ulrich B. D. (2012). Longitudinal changes in muscle activity during infants' treadmill stepping. J. Neurophysiol. 108, 853–862. 10.1152/jn.01037.2011 22490560 PMC3424088

[B61] Teulier C. Ulrich B. D. Martin B. (2011). Functioning of peripheral Ia pathways in infants with typical development: responses in antagonist muscle pairs. Exp. Brain Res. 208, 581–593. 10.1007/s00221-010-2506-x 21140137 PMC4375015

[B62] Thelen E. Fisher D. M. (1983). The organization of spontaneous leg movements in newborn infants. J. Mot. Behav. 15, 353–377. 10.1080/00222895.1983.10735305 15151867

[B63] Tresch M. C. Kiehn O. (2002). Synchronization of motor neurons during locomotion in the neonatal rat: predictors and mechanisms. J. Neurosci. 22, 9997–10008. 10.1523/JNEUROSCI.22-22-09997.2002 12427857 PMC6757827

[B64] von Hofsten C. (2004). An action perspective on motor development. Trends Cogn Sci. 8, 266–272. 10.1016/j.tics.2004.04.002 15165552

[B65] Walsh E. G. (1976). Shortening reactions in the human forearm. J. Physiol. 256:116.

[B66] Westerga J. Gramsbergen A. (1994). Development of the EMG of the soleus muscle in the rat. Brain Res. Dev. Brain Res. 80, 233–243. 10.1016/0165-3806(94)90108-2 7955348

[B67] Westphal C. (1880). Ueber eine Art paradoxer Muskelcontraction. Arch. Psychiatr. 10, 243–248. 10.1007/BF02224565

[B68] Xia R. Sun J. Threlkeld A. J. (2009). Analysis of interactive effect of stretch reflex and shortening reaction on rigidity in Parkinson's disease. Clin. Neurophysiol. 120, 1400–1407. 10.1016/j.clinph.2009.05.001 19487158

[B69] Yang J. F. Mitton M. Musselman K. E. Patrick S. K. Tajino J. (2015). Characteristics of the developing human locomotor system: similarities to other mammals. Dev. Psychobiol. 57, 397–408. 10.1002/dev.21289 25754858

